# E-quality Control in Dental Metal Additive Manufacturing Inspection Using 3D Scanning and 3D Measurement

**DOI:** 10.3389/fbioe.2020.01038

**Published:** 2020-08-27

**Authors:** Liang Du, Yiwen Lai, Chunwang Luo, Yong Zhang, Jun Zheng, Xiaohong Ge, Yuangang Liu

**Affiliations:** ^1^School of Electrical Engineering and Automation, Xiamen University of Technology, Xiamen, China; ^2^School of Materials Science and Engineering, Xiamen University of Technology, Xiamen, China; ^3^College of Chemical Engineering, Huaqiao University, Xiamen, China

**Keywords:** E-quality, dental restoration, metal materials, 3D scanning, 3D measurement

## Abstract

3D printed metal crowns can be used for dental restorations. The main quality control challenge of these dental metal is the method of quality inspection. Electronic quality is a process by which the quality of the process and the parts produced can be checked online, thereby improving the process and reducing the time it takes for the entire process. Here, we propose a combination of 3D scanning and 3D measurement for 3D inspection of metal crowns. The data extracted from the 3D printed metal crowns were used as case studies to prove the proposed methodology. The obtained results confirm that the new method has very high classification accuracy compared with the traditional inspection methods, and thus yields excellent results. Moreover, the proposed approach is capable to archive 3D models of the parts and achieve rapid quality control. This paper forms the basis for solving many other similar problems that occur in 3D printing related industries.

## Introduction

Selective laser melting (SLM) technology can quickly prepare metal crowns suitable for the individual needs of different patients (Huang et al., 2015; Buican et al., 2017). However, the choice of different materials and the setting of process parameters may cause defects in printed crowns. In this case, the E-quality control system was developed which is benefit to perform quality inspections reliably, accurately and in a very short time (Liverani et al., 2017).

Traditional methods to achieve and ensure quality standards are mainly through statistical process control (SPC) procedures (Zhai et al., 2004). However, the product quality in the sequential manufacturing process is affected by various factors, and it is difficult to set the optimal manufacturing specifications for SPC (Kang et al., 1999). Traditional SPC and six sigma techniques must adhere to statistical assumptions such as the normality of the variable distribution, constant variance of the variables, and so on. However, it is difficult to satisfy all these assumptions in practice. Based on some shortcomings of current statistical methods, a hybrid data mining method based on the combination of rough set theory, fuzzy set theory, genetic algorithm and agent based technology is proposed. Compared with standard statistical tools that use population based methods, the Rough Set Theory (RST) uses an individual approach based on an object model, which is a very useful tool for analyzing quality control issues (Kusiak, 2001). In addition, Fuzzy Set Theory (FST) has proven its function in many applications, especially for the control of complex non-linear systems that may be difficult to model analytically (Huang et al., 2008). The Genetic algorithm (GA) is based on the overall solution rather than a single solution (Goldberg, 1989). In order to solve the shortcomings of these statistical methodologies in the quality control of dental metal, the proposed approach expects to provide a way to optimize prediction for the lowest defective rate.

Recent work has shown that 3D dental models can be obtained through 3D scanning, which can be used for rapid prototyping, parts inspection and dimensional control (Munera et al., 2012; Javaid et al., 2019). This type of technology can use light as a probe, which does not damage the sample during analysis, ensuring that the measurement technology will not deform the object (Zhang and Alemzadeh, 2007). Therefore, it is of great significance to obtain 3D reconstruction of dental models. However, the related research mainly focused on reconstruction and measurement of plaster dental models or casting dental models, while there are few studies on dental metal models manufactured by SLM (Sinescu et al., 2008; Zhou and Fan, 2017; Song et al., 2018; Yang et al., 2018). We already have a case of E-quality control of automotive parts, but we still face some challenges for the E-quality control of metal crowns manufactured by SLM.

This paper presents a 3D E-quality control system that integrates 3D scanning and 3D reconstruction to perform fast quality control inspections of complex dental metal parts. The systems then compare the scanned 3D models of produced parts with the scanned 3D models of the standard parts to provide accurate and timely measurement feedback for quality control, thereby helping to prove that the produced products meet the required specifications.

## Methodology

### The 3D E-quality Control System

**FIGURE 1:**
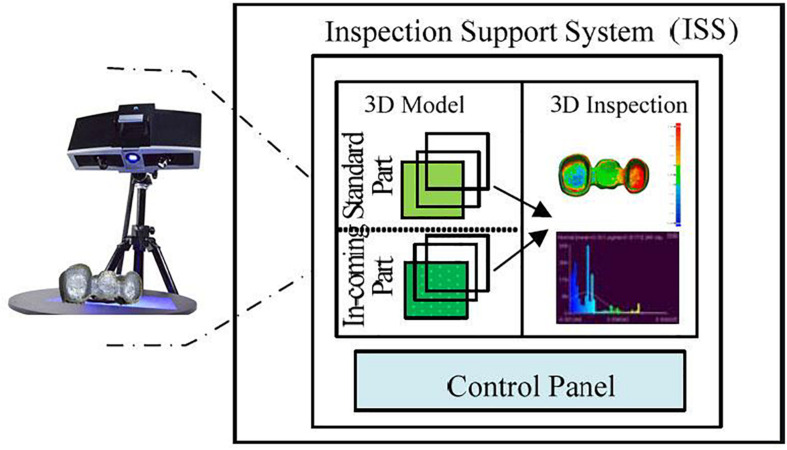
The framework of the 3D E-quality control systems.

The 3D E-quality control system advances the basic research to address the aforementioned issues through the use of integration of cyber communication, virtual prototyping, 3D scanning, and machine vision system. The hardware is mainly composed of the 3D scanning system and the machine vision system. These equipment and systems provide the foundation for the project. The software consists of Intelligent Graphical User Interface (i.e., Application Program Interface) embedded with 3D reconstruction and 3D measurement algorithms. The overall framework of the 3D E-quality control system is shown in Figure 1. Firstly, the inspection support system (ISS) uses a 3D scanner to scan the metal crowns to generate high-resolution 2D images and accurate 3D models for inspection. The 3D scanning System based on Structured light is used to capture high-resolution 2D images of parts from different angles with blue light patterns. After these images have been produced, they are used to construct accurate 3D models which will be saved in the PC and compared with the standard 3D model later for the comparison is implemented, the contrast and difference between two parts will be identified. Finally, the inspection outcomes will be reported as Pass, Rework, and Discard.

### 3D Reconstruction

**FIGURE 2:**
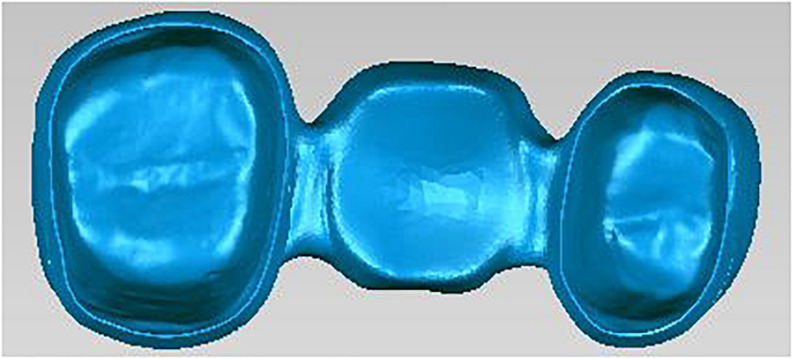
Scanned geometry of metal crowns.

The scanning process of metal crowns is carried out by using a 3D scanner. The scanner is the medium of data capture. The purpose of the 3D scanner is to measure and record the relative distance between the surface of the object and a known point in space. This geometric data is expressed in the form of point cloud data (Sinescu et al., 2008). In order to generate accurate 3D models for inspection, 3D reconstruction of metal crowns is required. The structured light used in the scanning process is blue, and the light pattern consists of a series of stripes. In capturing, the grating projection device projects several pieces of structured light with a specific code onto the metal crowns, and the two cameras at a certain angle simultaneously acquire corresponding images. Because of noise and limited resolution of the projector, the scanning of structured light will introduce some high frequency offset and noise. We use bilateral noise reduction to smooth the structured light scanning surface, then create a surface normal maps from the smoothed grid, and use high-pass filtering to extract the high frequency details of the estimated normals (Mallick et al., 2005) (see Figure 2).

Finally, we use the embossing process in Zickler et al. (2002) to optimize the mesh vertices to match this combined normal map. We obtain diffuse and specular normals from gradient illumination. For the polarization pattern, a separate linear polarizer is placed on each light. The linear polarizer is installed on the servo motor in front of the camera, which can quickly flip the polarizer between its horizontal and vertical directions at its diagonal (Mallick et al., 2005).

While using normals to improve the geometry, we found the measured position from the distance image. Reference to the pixel coordinates on the camera will cause the natural parameterization of the corresponding surface. Therefore, under perspective projection, the coordinates of the surface points can be represented by the depth function Z (x, y). That is to say, given the pixel coordinates, the position of the corresponding surface point P (x, y) has only one degree of freedom Z (x, y):

(1) $$P\,\left(x,y\right)={\left[-\frac{x}{f_{x}}\,Z\,\left(x,y\right),-\frac{y}{f_{y}}\,Z\,\left(x,y\right),-\frac{x}{f_{x}}\,Z\,\left(x,y\right)\right]}^{T}$$

Where f_x_ and f_y_ are the focal length of the camera in pixels. The challenge we face is to find a depth function that matches our estimate of the position and normal of each point. To this end, we select a depth function to minimize the sum of two error terms: position error E^p^ and normal error E^n^. Then define the position error as the sum of the square of the distance between the optimized position and the measured position:

(2) $${||P_{i}-P_{j}^{m}||}^{2}=\mu_{i}^{2}\,{\left(Z_{i}-Z_{i}^{m}\right)}^{2},w\,h\,e\,r\,e\,\mu_{i}^{2}={\left(\frac{x_{i}}{f_{x}}\right)}^{2}+{\left(\frac{y_{i}}{f_{y}}\right)}^{2}+1$$

The surface tangents T_x_ and T_y_ at a given pixel can be expressed as a linear function of the depth value and its partial derivative:

(3) $$T_{x}=\frac{\partial P}{\partial x}={\left[-\frac{1}{f_{x}}\,\left(x\,\frac{\partial Z}{\partial x}+Z\right),-\frac{1}{f_{x}}\,y\,\frac{\partial Z}{\partial x},\frac{\partial Z}{\partial x}\right]}^{T}$$

(4) $$T_{y}=\frac{\partial P}{\partial y}={\left[-\frac{1}{f_{x}}\,x\,\frac{\partial Z}{\partial y},-\frac{1}{f_{y}}\,\left(y\,\frac{\partial Z}{\partial y}+Z\right),\frac{\partial Z}{\partial y}\right]}^{T}$$

Then define the normal error as

(5) $$E^{n}=\sum_{i}{\left[T_{x}\,\left(P_{i}\right)\cdot N_{i}^{c}\right]}^{2}+{\left[T_{y}\,\left(P_{i}\right)\cdot N_{i}^{c}\right]}^{2}$$

The best surface is as follows:

(6) $$\arg\,\min_{Z}\gamma \,E^{p}+\left(1-\gamma \right)\,E^{n}$$

where the parameter γ ∈ [0,1] controls the degree of influence of position and normals in optimization.

### 3D Measurement Application Programming Interface (API)

**FIGURE 3:**
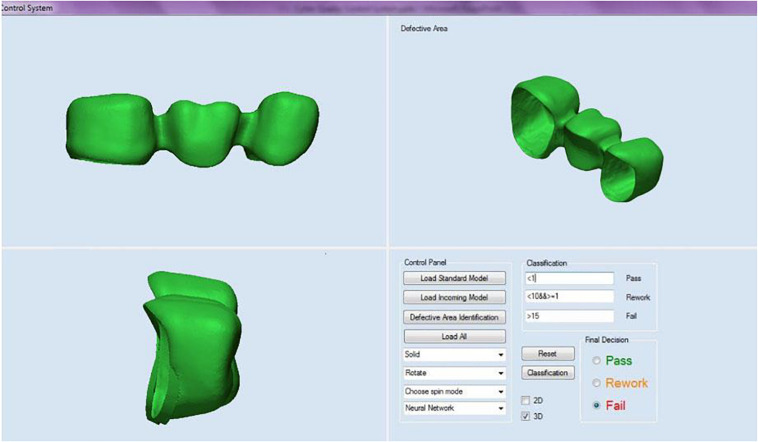
3D quality control API.

**FIGURE 4:**
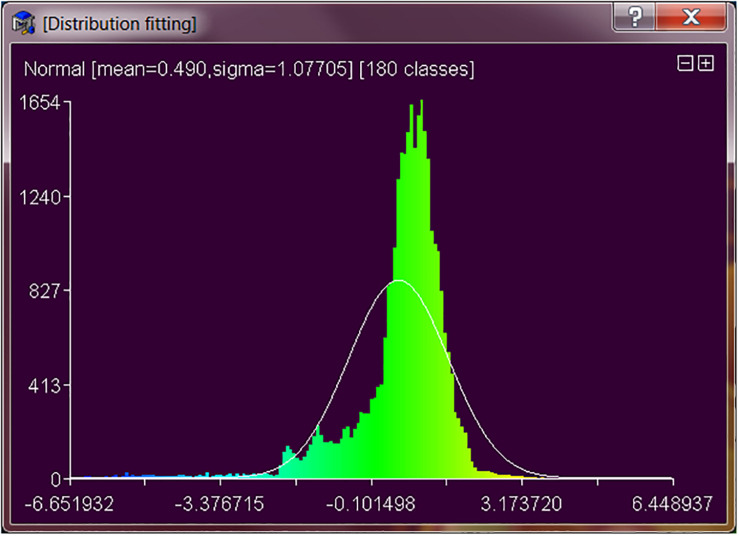
Distribution from 3D measurement.

Remote access can be achieved through an application programming interface (API). The purpose of API development is to provide a graphical user interface (GUI) to allow the user to establish and control communication lines with Web-enabled devices. As a result, the API allows the user to view, measure or operate dental metal parts through the machine vision system (MVS), webcam, and “remote desktop” provided by Microsoft^®^ Windows. The current 3D measurement API using point clouds to perform 3D inspections to measure the geometric elements of metal crown parts, such as contours, surfaces, boundaries, etc. It extracts features directly from the point cloud and provides feedback such as standard deviation (mean error), tolerance and distribution. Also it gets color mapping from surfaces or contours comparison and label edition on particular points for inspection. The inspector then can classify the part into Pass or Fail categories. The 2D inspection function can show the inspector the high-resolution images taken during the scanning process that are used to generate the accurate 3D model. The 2D inspection function can also lead the inspector to the Machine Vision System (MVS) for traditional 2D inspection (see Figures 3, 4).

### Registration of 3D Measurement

**FIGURE 5:**
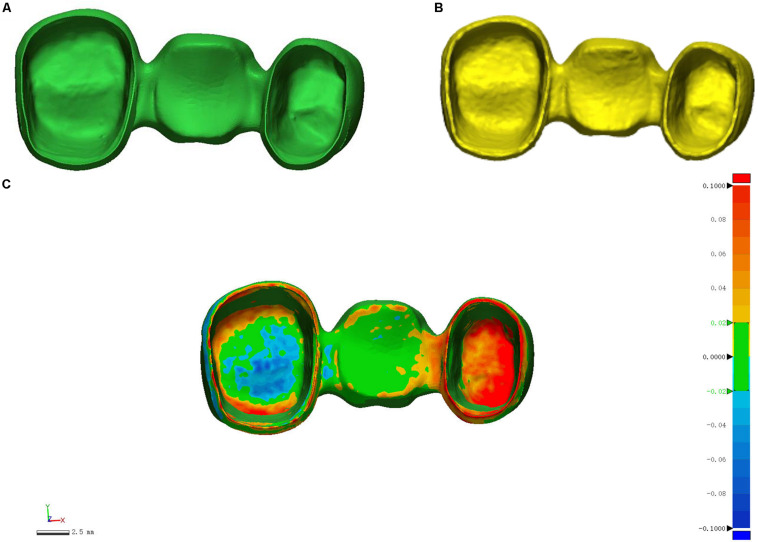
Register the two metal crown models before 3D measurement using the ICP algorithm. **(A)** Standard metal crown model; **(B)** Scanned metal crown model; **(C)** Registered metal crown models.

Before doing 3D measurement, we need to register the two models to be compared. The first step is to scale the two models to make sure they are expressed in the same units. First, select an accurate element that is visible in the two metal crown models, such as edges, surfaces, or other elements. Then, measure the distance D_*max*_ between two specific points on the model on a larger scale, and then repeat the process on the other model, and obtain the corresponding distance D_*min*_. Finally, the scale factor S_f_ = D_*max*_/D_*min*_ is calculated and applied to the smaller model in the f_x_, f_y_, and f_z_ fields of the 3D measurement API modification tool. In the second step, we roughly register the two models with translate and rotation tool of the API, and then use the Iterative Closest Point (ICP) algorithm to precisely register and finish the superimposition of the two models (Figure 5).

**FIGURE 6:**
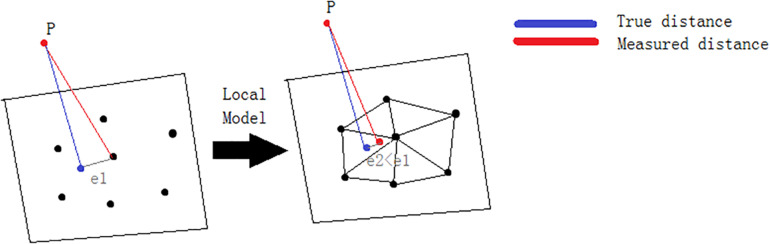
A local model is used to calculate the distance between two point clouds.

The default method for calculating the distance between two point clouds is “nearest neighbor distance.” For each point of the compared cloud, the API searches for the closest point in the reference cloud and calculates their (Euclidean) distance. If the reference point cloud is dense enough, an approximation of the distance from the comparison cloud to the base surface represented by the reference cloud can be accepted. However, if the reference cloud is not dense enough, the nearest neighbor distance may sometimes be inaccurate. Therefore, the 3D measurement API uses an intermediate method that can better approximate the true distance of the reference surface. When determining the closest point in the reference cloud, the idea is to locally model the reference cloud surface by fitting a mathematical model, e.g., Delaunay triangulation, located at the “nearest” point and its neighbors. The distance from each point in the comparison cloud to the closest point in the reference cloud will be replaced by the distance to the model (Figure 6). Statistically, this is more accurate and less dependent on cloud sampling.

## Experiment and Analysis

This case study involves the occurrence of two common defects in dental crown parts printed by SLM technology and the change on dimensions after forming. If the minimum strength requirements are not met, metallurgical defects may cause parts to crack. These requirements may be accomplished, but if appearance becomes the main requirement, they may be affected by such defects. Shrinkage is an important aspect to consider in the metal powder forming process, as it is essential for good fit and function of the final product. The final inspection of the process includes a visual inspection and a first inspection by the person in charge. Coordinate measuring machines may also be used to ensure dimensional accuracy of the final product. If defects are found, parts may be repaired manually, or in case if it cannot be repaired, it must be scrapped. Defects may occur randomly or simultaneously due to many factors. Therefore, checks are required to ensure that there are no other issues either with the forming process parameters, the materials or equipment used. If dimensional accuracy is not accomplished, parts are simply scrapped.

Selective laser melting process is a method of directly forming metal components, which has been widely used in medical, automotive, aerospace and other industries (Yadroitsev and Smurov, 2010; Hedberg et al., 2014). In the medical field, metal crowns are made of metal powder materials, usually cobalt chromium alloys or titanium alloys, depending on the characteristics and specifications of the materials (Averyanova et al., 2011; Xu et al., 2014). When designing dental parts, it must be very carefully designed for the patient to wear. Before this process, the small features of the parts, powder materials, process parameters, are some of the important points to take into account (Delgado et al., 2012; Zhang et al., 2012).

**FIGURE 7:**
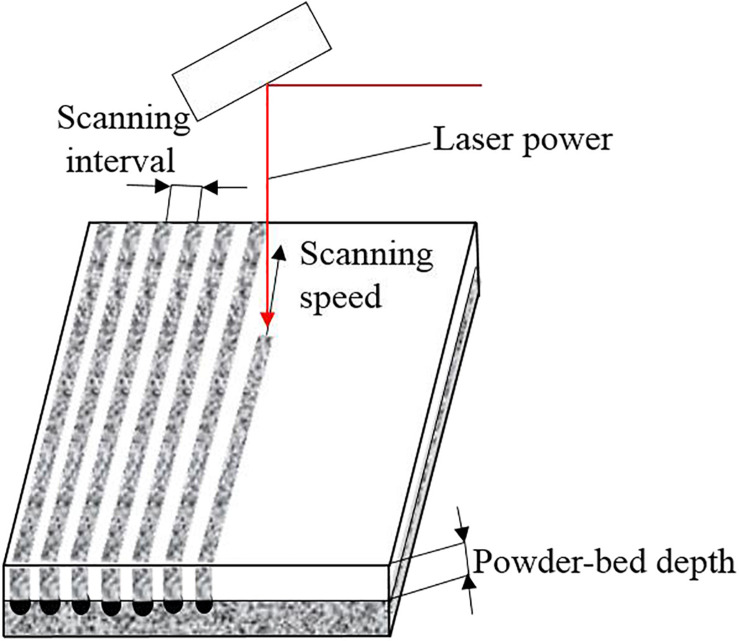
Key process parameters affecting metal crowns quality in SLM.

The key process parameters affecting the quality of metal crowns in SLM are shown in Figure 7. Laser power has an important influence on the quality of metal powder melting during rapid forming. If laser power is too low, the energy irradiated on the metal powder will be small, resulting in the powder cannot be completely melted, causing defects such as spheroidization and pores. However, if laser power is too high, excessive melting will occur, resulting in warping, deformation and cracking of the formed parts, which will have an adverse effect on the forming quality (Zhang et al., 2017). Scanning speed refers to the moving speed of the laser spot on the powder bed, which directly affects the interaction time between laser and metal powder. Scanning interval refers to the distance between two adjacent parallel scan lines, which determines the overlap between melt channels. Smallar powder-bed depth is generally beneficial to improve the forming quality, but at the cost of reducing processing efficiency. These important parameters are related to energy density, reflecting the complexity and difficulty of SLM process parameters control (Cherry et al., 2015).

When setting different process parameters to print dental parts, some defects may appear in the process. At this point, all employees involved in the process are trained to identify these issues. This means that quality control is dependent on a human being been able to identify such defects. Depending on the defect, the part may not meet its requirements and may not function as expected. There may be little details easily fixable, but there may be other details that cannot be resolved at all.

**FIGURE 8:**
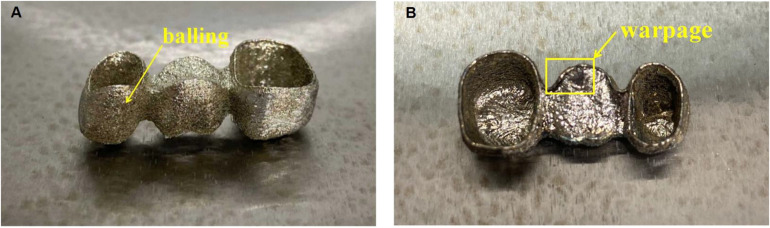
Cosmetic defects: **(A)** Balling, **(B)** Warpage.

Some appearance defects may include spheroidization, warpage, shrinkage, cracking, among others (Kang and Ma, 2017). We will focus on two of cosmetic defects: balling and warpage (Figure 8).

Since the laser melting channel has a tendency to reduce the surface energy under the action of surface tension, it is easy to form a balling phenomenon in the SLM process (Dai and Gu, 2015). The balling phenomenon is mainly caused by too low and too high energy density. The spheroidization on the sintering surface during SLM processing is the main reason for the existence of pores. The best energy density can be obtained by adjusting the process parameters so as to reduce the porosity of the sintered surface (Li et al., 2012; Galy et al., 2018).

Shrinkage and warpage are caused by residual stress (Liu et al., 2016). Such defects can be avoided by reducing the generation of residual stresses. Residual stress is an inevitable metallurgical defect when SLM processing metal parts. The main reason for its existence is the high temperature gradient during processing, and preheating the substrate can reduce residual stress (Salmi et al., 2017). At the same time, the warpage of parts can be reduced by optimizing the support structure and process parameters.

If a good quality control plan is not established and personnel are not properly trained to detect defective parts in a timely manner, scrapped parts may waste a lot of time and cost. It is required to find any defects before the parts are delivered or used so that they can be repaired, reworked or scrapped.

In order to solve the problem of surface metallurgical defects, some points need to be clarified (Olakanmi et al., 2015; Taib et al., 2016). First, you should check the exact location and shape of the defect and find out when it is really obvious. Then, we need to specify if the defect occurs once every time or irregularly, if it is always exists at a certain position of the metal crowns, if we can predict the defect and if it only happens with one 3D printer or others. If we are capable to identify defects on time, we can ensure that appropriate measures are taken to avoid major losses due to insufficient information.

To demonstrate the proposed methodologies of E-quality control in an industrial setting, metal crowns containing different types of defects including spheroidization, holes, cracking, and warpage were used for testing. This part requires certain specifications about size and durability. If it does not meet these specifications, such as cracking or deformation, it must be scrapped. If the dimensional accuracy of metal crown parts does not meet the requirements, it will not be suitable for patients to wear and cause discomfort. It is necessary to investigate what causes this problem. It depends on what kind of defects the parts have so as to make targeted technical improvements.

### Design of Experiments

**FIGURE 9:**
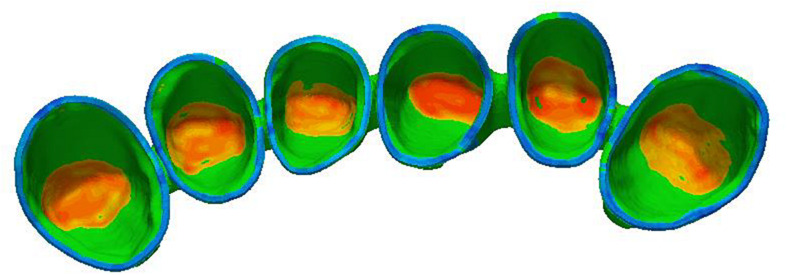
The green surface in the half-bridge crown is the key area for quality control.

A half-bridge crown model is shown in Figure 9. These parts have a tolerance limit of ± 0.25 mm. Parts with dimensions outside this range will be rejected. The green surface is important because the green surface is mainly the internal surface profile of the metal crowns.

**FIGURE 10:**
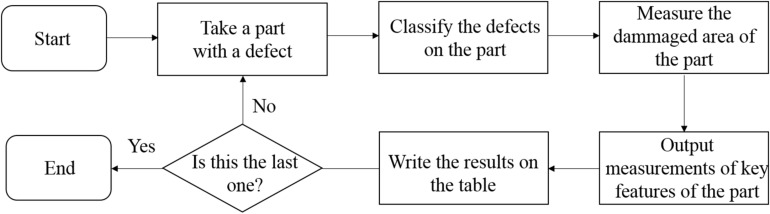
3D printed metal crowns quality control flowchart.

**FIGURE 11:**
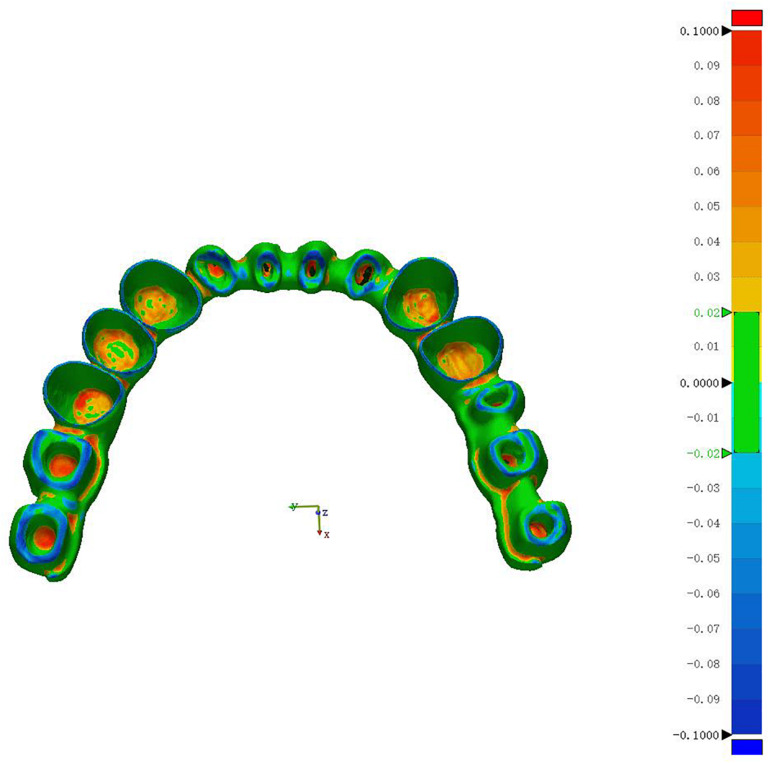
3D Measurement of dental metal parts.

Twenty metal crown parts were printed on the WXL-120 SLM equipment, and some of them exceeded tolerance limits for each size. Each type of dental metal part is mixed together for inspection. The test flow chart is shown in Figure 10 below. Although these parts does not pose any serious measurement challenges, it presents a moderate level of complexity for the requirement of our work. The 3D measurement of dental metal parts is shown in Figure 11.

### Classification Analysis

The following tables show the comparison of the classification analysis using 2D Machine Vision System, 3D Quality Control System and the efficiency test results. From Table 1 and Table 2, we can see that the 2D Machine Vision System can achieve accuracy of 0.85 and with precision of 0.875 while the 3D Quality Control System can achieve both accuracy and precision of 1 compared with the actual efficiency test results. With 3D E-quality control, all 20 dental metal parts are predicted correctly, resulting in 100% classification accuracy.

**TABLE 1:** The confusion matrix of the 2D Machine Vision System (MVS) classification results.

	Predicted	
Actual Efficiency Test	Pass	Fail
Pass	14	2
Fail	1	3
		

**TABLE 2:** The confusion matrix of the 3D Quality Control System (QCS) classification results.

	Predicted	
Actual Efficiency Test	Pass	Fail
Pass	16	0
Fail	0	4

## Conclusion

This paper proposes a 3D E-quality control system, which integrates 3D scanning and 3D reconstruction functions, and can perform rapid quality control inspection on complex metal crown parts manufactured by SLM. The scanner based on structured light can provide traceable data and accurate 3D models of 3D printed complex parts, thereby providing manufacturers with proof of compliance. The system captures millions of data points in just a few minutes to represent the true and complete geometry of complex metal crown parts. The systems then compare the scanned 3D models to computer aided design (CAD) models to provide accurate and timely measurement feedback for quality control, thereby helping to prove that the produced products meet the required specifications. The data extracted from the 3D printed metal crowns was used as a case study to prove the proposed methodology. The results show that, compared with traditional detection approach, this approach has better effect and higher classification accuracy. Through this approach, the following conclusions can be drawn: (1) Measuring the whole part can better ensure that the requirements are met and improve overall quality. (2) Scan and store 3D models of complex parts for technicians to view, analyze and measure later. (3) Create accurate digital models of existing components for redesign. (4) Help replicate complex parts, tooling or parts that are no longer produced.

## Data Availability Statement

The raw data supporting the conclusions of this article will be made available by the authors, without undue reservation.

## Author Contributions

All the authors were involved in this work. JZ and XG conceived the idea of the study. CL and YZ designed and performed the experiments. YLi helped with the experiments and provided constructive discussions. JZ provided the financially supporting for this work. YLa analyzed the data. LD, JZ, and YLi interpreted the data and wrote the manuscript. All authors contributed to the article and approved the submitted version.

## Conflict of Interest

The authors declare that the research was conducted in the absence of any commercial or financial relationships that could be construed as a potential conflict of interest.
